# A Case Report of Undiagnosed Chronic Constipation With Considerable Stool Burden

**DOI:** 10.7759/cureus.81914

**Published:** 2025-04-08

**Authors:** Andrej M Sodoma, Nicholas J Knott, James R Pellegrini, Scarlett Alvarez, Mary Thomas

**Affiliations:** 1 Internal Medicine, South Shore University Hospital, Bay Shore, USA; 2 Osteopathic Medicine, New York Institute of Technology, Jonesboro, USA; 3 Internal Medicine, Nassau University Medical Center, East Meadow, USA; 4 Gastroenterology, South Shore University Hospital, Bay Shore, USA

**Keywords:** : constipation, fecal disimpaction, gastrointestinal motility, ibs-c, obstipation

## Abstract

Chronic constipation is a prevalent issue in the United States that a majority of people have experienced at some time, with a variety of severity. Sometimes, people can become constipated for prolonged periods for various reasons. This is detrimental to one's health because the stool can build up in the colon and harden, creating obstipation. A 25-year-old female with an extensive history of chronic constipation and a history of hospitalization requiring medical management presented to the hospital with a chief complaint of minimal passage of stool over the last four months. Her vitals and labs were within normal limits. However, on physical exam, her abdomen felt like clay. A computed tomography (CT) scan showed a significant stool burden. The patient declined total colectomy with end ileostomy. She went for manual disimpaction under anesthesia, where they were able to evacuate stool up to the distal sigmoid colon. Following the procedure, the patient adhered to a pure liquid diet and a bowel regimen. Over time, the patient improved and was discharged on Miralax twice daily, with instructions to follow up with Gastroenterology and Colorectal Surgery for further monitoring. However, the patient did not follow up. A stool burden of this caliber is rarely seen. In this rare case, the patient resumed her everyday life with only medical treatment and endoscopy, offering a commentary on the current standard of care. Indeed, removing the aganglionic segment is the ultimate solution. However, surgery could potentially wait if the patient improves in this manner.

## Introduction

Chronic constipation is characterized by unsatisfactory defecation, difficulty with defecation, and unsatisfactory stooling [1]. Chronic constipation ranges from 9% to 20% of Americans, which is a highly prevalent problem that the majority of people have experienced at some time [2,3]. The Rome IV criteria say someone has it if two of the following six conditions are met. The symptoms have been present for three months in more than 25% of bowel movements: straining while defecating, hard stools, the feeling of incomplete evacuation, the sense of an anorectal blockage, having to do manual maneuvers to defecate, and having less than three bowel movements per week. The constipation severity index measures constipation as mild (less than three bowel movements per week with minimal discomfort), moderate (one to two strenuous bowel movements per week with abdominal pain), and severe (less than one per week and can impact the quality of life) [4].

Sometimes, when the stool burden builds up enough, the stool hardens, creating obstipation and the inability to pass stool or gas. Obstipation can be a result of several pathologic factors, including trauma, surgery, electrolyte imbalance, chronic constipation, and obstruction causing colonic inertia (CI). CI is a severe type of chronic constipation with significantly slowed stool transit, affecting 5% to 30% of patients with slow-transit constipation [5]. With this broad differential, understanding the underlying etiology is prudent for management, as it can vary from noninvasive medical management to invasive surgical management. In this case report, a patient came in with a history of severe constipation with a significant stool burden. The standard of care for these cases is definitive therapy with surgical removal of the affected portion of the colon. However, the patient refused treatment, opting for medical interventions only, with evident improvement and resumption of everyday life.

## Case presentation

A 25-year-old female presented to the hospital for abdominal pain and lack of bowel movement for four months. She has a history of chronic constipation, starting around the time she was toilet-trained. Besides imaging to confirm the etiology of the abdominal pain, which would be revealed as a significant stool burden, no manometry or biopsy was performed for the diagnosis of the root cause of the constipation. She needed weekly enemas and daily stool softeners, with multiple hospital visits for manual disimpaction in the operating room (OR) under anesthesia before this admission. After the most recent hospital stay a few years ago, the patient followed up with Gastroenterology, who prescribed a bowel regimen of polyethylene glycol 3350. Her constipation was improving, so over a year, her treatment was adjusted from taking a gallon of GoLYTELY (polyethylene glycol 3350) daily to one packet of Miralax (polyethylene glycol 3350) per day. The most recent visit with her gastroenterologist was seven months before her current hospital admission. During that visit, her Miralax dosage was reduced from two packets to one packet per day. After the dosage change, she began intermittently skipping doses and stopped taking Miralax altogether because she did not see the importance of taking a single packet of Miralax daily.

When presenting to the hospital, the patient endorsed minimal passage of stool in the last four months. She experienced occasional involuntary leakage of watery stool every few weeks. The patient never returned to the previous regimen of Miralax or revisited Gastroenterology due to societal obligations. A few weeks before admission, the patient began to experience abdominal pain and abdominal distension, not responsive to over-the-counter stool softeners, stimulant laxatives, and enemas, prompting the patient's visit to the emergency department (ED). 

**Figure 1:**
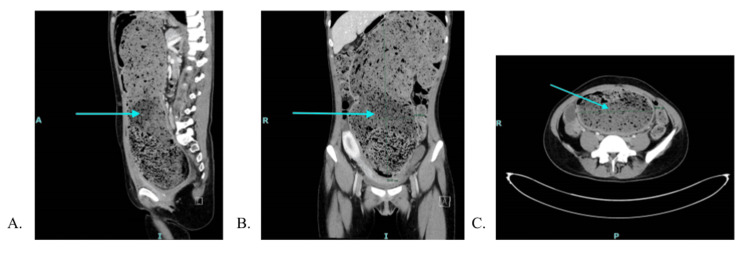
CT abdomen/pelvis with IV contrast on the day of admission, demonstrating a distended colon A: Sagittal view. B: Coronal view. C: Transverse view. The blue arrow points at the most distended portion of the colon.

On arrival, the patient was vitally stable (Table 1). Labs showed a complete blood count within normal limits (Table 2) and a basic metabolic panel within normal limits (Table 3). On physical exam, the patient's abdomen was firm with generalized tenderness but no rebound tenderness. The left lower quadrant (LLQ) and right lower quadrant (RLQ) felt like clay. When pressing, the practitioner could leave indents in the abdomen due to the stool burden. A rectal exam was performed, and no hemorrhoids/fissures were visualized. Adequate rectal tone and hard, dark brown stool in the rectal vault. A computed tomography (CT) scan of the abdomen and pelvis was ordered. It showed a 15-cm-diameter redundant sigmoid colon that was stretched out and had a lot of stool in it all along the GI tract (Figure 1).

**Table 1:** Complete blood count with differential on admission

Complete blood count with differential		Normal range
White blood cell count (cells/uL)	6,460	4,000-11,000
Red blood cell count (M/uL)	4.69	3.80-5.20
Hemoglobin (g/dL)	12.9	12-15.5 (women)
Hematocrit (%)	41	36-48 (women)
Mean cell volume (fL)	87.4	80-100
Mean cell hemoglobin (pg)	27.5	27-34
Mean cell hemoglobin concentration (g/dL)	32	32-26
Red cell distribution width (%)	13.3	10.3-14.5
Platelet count (mcl)	266,000	150,000-450,000
Neutrophil automated count (K/uL)	3.83	1.80-7.40
Lymphocyte automated count (K/uL)	2.07	1.00-3.30
Monocyte automated count (K/uL)	0.39	0.00-0.90
Eosinophil automated count (K/uL)	0.12	0.00-0.50
Neutrophil automated percent	59.3	43-77
Lymphocyte automated percent	32.0	13-44
Monocyte automated percent	6.0	2-14
Eosinophil automated percent	1.9	0-6
Basophil automated percent	0.6	0-2
Immature granulocyte automated percent	0.2	0.0-0.9

**Table 2:** Vital signs on admission with normal value ranges

Vitals		Normal range
Blood pressure (mmHg)	114/77	<120/<80
Heart rate (bpm)	84	60-100
Respiratory rate (rpm)	20	12-20
Temperature (F)	98.4	97-99
Oxygen saturation (%)	97	95-100

**Table 3:** Basic metabolic panel on admission with normal ranges

Basic metabolic panel		Normal range
Sodium (meq/L)	139	135-145
Potassium (meq/L)	4.5	3.5-4.5
Chloride (meq/L)	106	96-106
Bicarbonate (meq/L)	22	22-29
Anion gap (meq/L)	12	4-12
Blood urea nitrogen (mg/dL)	9.8	6-24
Creatinine (mg/dL)	0.62	0.5-1.1
Glucose (mg/dL)	88	<140 (random)

We consulted Gastroenterology and Colorectal Surgery about disimpaction and the potential need for a colectomy. The patient declined enemas, suppositories, and manual disimpaction at the bedside due to severe discomfort and minimal to no results during previous hospitalizations. Colorectal Surgery offered a total colectomy with end ileostomy and a biopsy of the removed tissue to find out what was causing the constipation, but the patient turned it down.

**Figure 2:**
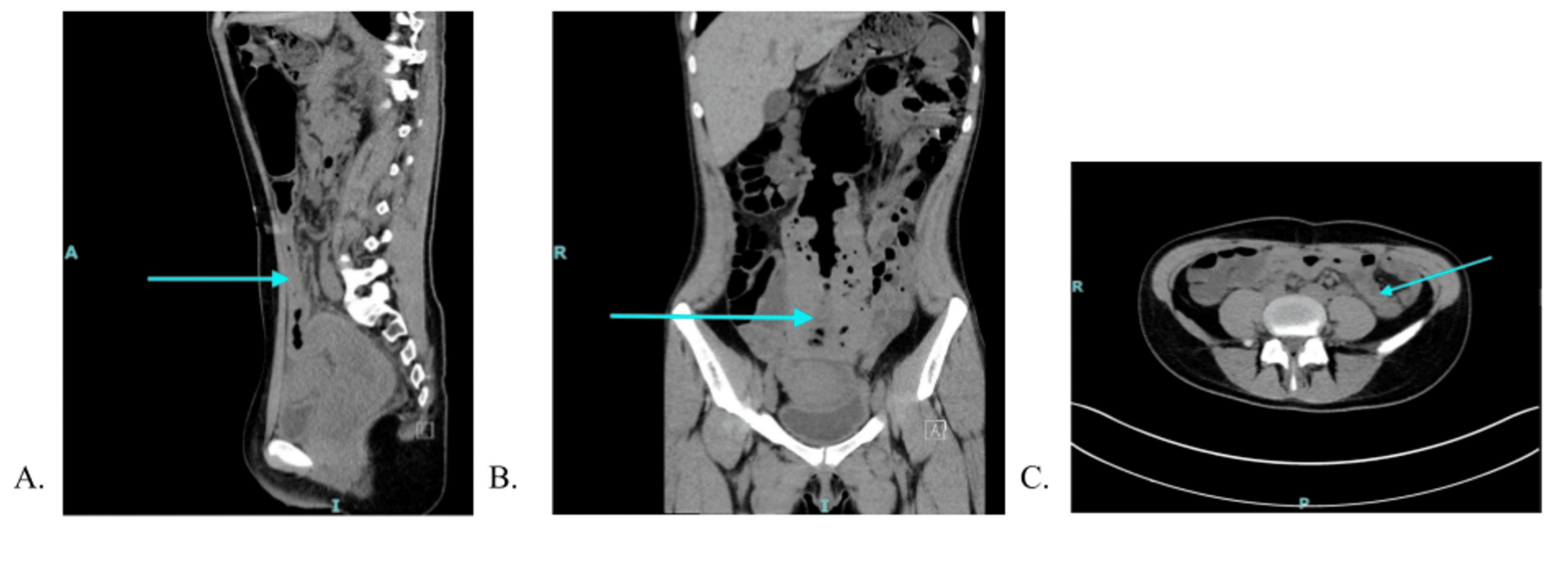
Repeat CT abdomen/pelvis with IV contrast on the day of discharge, demonstrating the colon cleared of stool A: Sagittal view. B: Coronal view. C: Transverse view. The blue arrow points at the formerly most distended portion of the colon post-medical and endoscopic management.

She went for manual disimpaction under anesthesia. Once the patient was anesthetized, the endoscopist manually disimpacted and then advanced the colonoscope into the center of the stool. The stool was then flushed with saline to loosen it and then dragged distally to remove it manually. The process was repeated multiple times up to the distal sigmoid colon. At that site, the endoscopist could not advance the colonoscope because of a lack of visibility as the stool was tough, and no amount of softening with lavage could move the stool. Also, the practitioner could not reach that area manually, so the procedure had to be stopped. During the procedure, it was noted that the colon was markedly dilated, and the stool resembled dense/moist clay. Following the procedure, the patient adhered to a pure liquid diet and a bowel regimen, including Miralax twice daily and a gallon of GoLYTELY daily. Her hospital course included a total recorded stool count of 21. Her admission weight was 128.9 lbs, and her discharge weight was 119 lbs. Before discharge, a CT scan of the abdomen was repeated to determine the extent of resolution (Figure 2). The patient was discharged on Miralax twice a day, with instructions to follow up with Gastroenterology and Colorectal Surgery for further monitoring. The patient followed up with Gastroenterology; her laxative regimen was tapered down further, and a repeat colonoscopy with biopsy was scheduled. Unfortunately, the patient did not attend the procedure and was lost to follow-up.

## Discussion

The patient discussed had a longstanding history of chronic constipation, which required daily bowel regimens that included laxatives, stool softeners, and enemas routinely since childhood to ensure a regular bowel movement. She stopped this regimen without resumption for four months, causing severe stool burden. The patient already had a long-standing history of daily motility agents, and once stopped, manual disimpaction was required, which is a hallmark of CI [6]. CI is rare and more severe than constipation, and CI is more common among older individuals [7]. It is more severe because it is an inherent issue with GI motility.

GI motility is performed by the interstitial cells of Cajal (ICC), which develop from the migration and maturation of neural crest cells [8]. Hirschsprung disease (HD) is described as the lack of neural crest migration, including ICC, to the distal colon, resulting in a malfunctioning segment and chronic constipation [9]. The lack of ICC is the most common histologic finding in patients with CI, suggesting an overlap in those with adult-onset HD, resulting in an overbearing stool burden [10]. Colonic pseudo-obstruction, whether acute or chronic, can present similarly, with an increased risk of ischemia and perforation as the colon dilates to catastrophic diameters [11]. The etiology of pseudo-obstruction has been described as myopathic, mesenchymopathic, and neuropathic, including loss of integrity of ICC, suggesting another overlapping condition leading to increased stool obstipation [12,13]. A detailed differential diagnosis is outlined in Table 4.

**Table 4:** Differential diagnosis

Differential diagnosis
Irritable bowel syndrome
Inflammatory bowel disease
Colonic inertia
Pelvic floor dyssynergia
Hirschsprung disease
Ogilvie syndrome
Intestinal pseudo-obstruction

While the patient declined the necessary workup for the diagnosis of CI and HD, her history and presentation place them high on the differential. A report of four cases of adult HD by Qiu et al. found that these patients presented with chronic constipation and/or intestinal obstruction. They usually have a history of chronic refractory constipation when, in reality, it is mild HD. The diagnosis is not made until a biopsy of the affected colon segment is obtained [13]. 

Necessary studies would be colonic motility studies and biopsies. Without proper persistent management, this patient will likely develop chronic megacolon resulting from colonic movement abnormalities [14]. This would increase her risk for perforation due to weakened colonic walls, recurrent episodes of obstipation, and chronic fecal soiling. The person will eventually need surgery to remove part of their colon and connect it to the ileum or rectum, or they may need a colostomy [15,16]. However, this was deferred due to the patient's age and the relative success of non-invasive medical therapies. The patient was educated to maintain the bowel regimen and have regular follow-ups with Gastroenterology. 

An article by Gamez et al., a case report and literature review, comprised case reports and case series of HD patients [17]. It consisted of a total of 36 HD patients. They commonly present with intestinal obstruction and fecal impaction. All of the cases had surgical intervention with either Duhamel, Saove, Swenson, or Rehbein procedures [17]. The patient in this case report received medical treatment, including endoscopy, per their preferences. Despite it straying from the standard of care, it is important and sometimes forgotten to consider the patient’s wishes. She was a young female who wanted to live without an ostomy bag for as long as possible. Upon further literature review, no cases of purely medical management for HD have been reported. This single case shows that the patient is resuming everyday life after a minimally invasive approach and offers a commentary on the current standard of care. Still, the loss of follow-up, with it being a single case, is a limitation. Indeed, removing dilated segments is the ultimate solution. However, if the patient improves in this manner, surgery could potentially be delayed. 

## Conclusions

This case investigates a unique treatment with a positive outcome of a severe case of colonic inertia. Overall, the standard of care is surgical removal of the dilated segment with multiple biopsies to understand the underlying etiology. However, the patient opted for medical management, which reduced the stool burden and allowed for the resumption of everyday life. This case offered a potential treatment plan for colonic intertia. In the future, further research must be done to examine this conservative measure compared to invasive surgical measures. 
